# Trend of Human Schistosomiasis Japonica Prevalence in China from 1990 to 2019

**DOI:** 10.3390/tropicalmed8070344

**Published:** 2023-06-29

**Authors:** Yifeng Li, Tingting He, Jingzi Xie, Shangbiao Lv, Zongguang Li, Min Yuan, Fei Hu, Dandan Lin

**Affiliations:** 1 Jiangxi Provincial Institute of Parasitic Diseases, Nanchang 330096, China; liyifeng1004@163.com (Y.L.); lvshangbiao2023@163.com (S.L.);; 2 Jiangxi Province Key Laboratory of Schistosomiasis Prevention and Control, Nanchang 330096, China

**Keywords:** Schistosomiasis japonica, prevalence rate, time trend, joinpoint regression, China

## Abstract

Schistosomiasis (Schistosomiasis japonica) remains an important public health problem in China, and the Chinese government has set an ambitious goal of eliminating schistosomiasis by 2030. Based on the observational study of the Global Burden of Disease Study database in 2019 (GBD2019) and the World Bank database, this study aimed to analyze the prevalence trend of schistosomiasis in China from 1990 to 2019 by using the joinpoint regression model, and the relationship between economic and social development and schistosomiasis prevalence. The data of age-standardized infection rates (ASRs) from the Global Burden of Disease Study Global Health Data Exchange were collected, and Gross national product per capita (GDP) and people using safely managed sanitation services ((PPMS) % of population) were extracted from the World Bank database. Trends of ASR were analyzed using joinpoint regression analysis, the association of ASR with GDP, and PPMS using the Pearson correlation analysis. The results reveal that, from 1990 to 2019, the overall trend of ASR from schistosomiasis showed a decrease for both sexes, the decreases in men were relatively smaller compared with women. A larger decrease has been observed in the age groups from 15 to 49 years compared with other age groups. The ASR of schistosomiasis had a significant negative correlation with GDP and PPMS. This observational study identified decreasing prevalence rate of schistosomiasis in China since 1990. Continuous investment, optimization of control strategy, and economic development will help to achieve the goal of schistosomiasis elimination.

## 1. Introduction

Schistosomiasis, caused by infection with blood-dwelling fluke worms of the genus *Schistosoma*, is considered a major public health problem in tropical and subtropical regions such as Africa, Asia, the Caribbean, and South America [1,2,3]. It is classified by the World Health Organization as one of 20 neglected tropical diseases [1]. It is estimated that schistosomiasis is transmitted in over 78 countries, with over 250 million people infected, and approximately 780 million people at risk of infection [4,5]. According to the Global Burden of Disease Study 2019, the global burden of schistosomiasis is estimated to be 1640 thousand disability-adjusted life years (DALYs) [6]. Some previous studies have estimated the global burden of schistosomiasis at 1700–4500 thousand DALYs [7,8].

There are six species of the parasite *Schistosoma* that infect humans: *Schistosoma mansoni*, *S. haematobium*, *S. intercalatum*, *S. guineensis*, *S. japonicum*, *and S. mekongi* [9]. China is one of the countries where *Schistosoma japonicum* (schistosomiasis for short) is transmitted. In the early 1950s, schistosomiasis was severely endemic in southern China, with more than 11.6 million schistosomiasis patients, 1.2 million infected cattle, and 14.8 billion square meters (m^2^) of snail areas in the whole country [10]. Since 1955, schistosomiasis control has been the subject of continuous investment by the Chinese government and national control programs [11]. After more than 60 years of struggle, the number of cases and infected livestock has decreased significantly [12]. However, schistosomiasis remains an important public health problem in China. By the end of 2021, there were 29,000 cases of advanced schistosomiasis and 3.69 billion square meters of snail-infested areas [13].

In China, schistosomiasis has been prevalent in 12 provinces of the Yangtze River Basin for more than 2100 years [10]. The schistosomiasis patients and infected cattle are the most important infectious source and the *Oncomelania hupensis* is the sole intermediate host of schistosomiasis. Schistosome infection occurs by contact with fresh water contaminated by cercariae released by the intermediate host snail [9]. The common symptoms of schistosomiasis are caused by the body’s reaction to the worms’ eggs, such as granuloma of liver and intestine. Schistosomiasis is also associated with undernutrition, exercise intolerance, diarrhea (sometimes bloody), chronic pain, and anemia [9]. “The Outline of the Healthy China 2030 Plan” clearly requires the whole country to achieve the goal of eliminating schistosomiasis before 2030. Some control measures are very important to achieve this goal, such as adjusting the agricultural industry structure to reduce cattle breeding, building sanitary toilets to reduce the pollution of worm eggs to the environment, increasing investment to carry out environmental transformation, and reducing the snail area.

Reviewing and analyzing the epidemic trend, change rule, and influencing factors of schistosomiasis in China is helpful to summarize the experience of prevention and control, promote the prevention and control of schistosomiasis, and provide experience for other countries [14]. In recent years, researchers in China have used time series analysis to analyze the epidemic trend of local schistosomiasis, which provides an important basis for the formulation of control measures [15,16,17,18,19]. However, the national schistosomiasis epidemic trend and its relationship with socioeconomic development and rural health from a long-term perspective has not been reported.

In this study, the joinpoint regression model was used to analyze the trends and patterns of schistosomiasis in China from 1990 to 2019, based on the observational study of the Global Burden of Disease Study database (GBD2019). In addition, based on the World Bank database, the relationship between indicators of economic and social development and schistosomiasis prevalence in China was analyzed. Based on these results and findings, this study proposes recommendations to optimize schistosomiasis control strategies and measures.

## 2. Methods

### 2.1. Study Design

This study adopts a descriptive method to investigate the prevalent trend of schistosomiasis in China from 1990 to 2019, as well as the relationship between socioeconomic development and schistosomiasis prevalence. Firstly, the data of age-standardized infection rates (ASRs) was extracted from Global Health Data Exchange (GHDx) to analyze the schistosomiasis prevalence trend from 1990 to 2019. In addition, data of Gross national product per capital (GDP) and people using safely managed sanitation services ((PPMS)% of population) were collected from World Bank database to examine the relationship between GDP, PPMS, and ASR. Data analysis includes both Joinpoint regression analysis and inferential analysis. Joinpoint regression analysis is used to explore the schistosomiasis prevalent trend, while inferential analysis, such as correlation analysis, is employed to examine the associations between GDP, PPMS, and ASR. This study systematically analyzes and interprets the trends in schistosomiasis prevalence in China over a 30-year period, providing valuable recommendations for control strategies.

### 2.2. Study Population

The infection rate data in this study were collected from the results of GDB2019, which were estimated by identifying multiple relevant data sources including health reports, disease notifications, and scientific literature, such as China Health Statistics Yearbook 2009 and China Notifiable Infectious Diseases 2008. The study population in those data sources is mainly consisted of residents aged 6 and above in 12 schistosomiasis-endemic provinces in China.

### 2.3. Data Source

The general methods for the GBD2019 and the methods for estimations of disease burden in schistosomiasis have been detailed in previous studies [6,20]. Annual prevalence rate and age-standardized prevalence rate (ASR) of schistosomiasis of China from 1990 to 2019, by sex and age, were collected from the Global Health Data Exchange (GHDx) query tool. For all age-standardized rates, GBD uses a standard population, which is calculated as the unweighted average across all countries of the percentage of the population in each 5-year age group for the years 2010–2035 from the World Population Prospects of the United Nations Population Division.

Gross national product per capita (GDP) and people using safely managed sanitation services ((PPSM) % of population), categorized by years (1990–2019), were extracted from the World Bank database (https://databank.worldbank.org/home.aspx, accessed on 19 October 2022). Due to the lack of data before 2000 and schistosomiasis transmitting in rural areas mostly, the PPMS was selected from 2000 to 2019 for rural areas. GDP is defined as the gross domestic product divided by the number of people in the year. PPMS is defined as the percentage of people using improved sanitation facilities that are not shared with other households and where excreta can be safely disposed of in situ or transported and disposed of offsite.

### 2.4. Statistical Analysis

The epidemic data of schistosomiasis in China from 1990 to 2019 were loaded into Microsoft Excel 2019 to establish a database. The difference between the start and end age-standardized prevalence rates (ASRs) for both men and women were calculated to determine the absolute and relative changes in ASR over the observation interval.

The Joinpoint software (Joinpoint Command Line version 4.9.0.1) was provided by the US National Cancer Institute Surveillance Research Program to assess the prevalence trend. The program starts with the minimum number of joinpoints (e.g., 0 joinpoints, which is a straight line) and tests whether more joinpoints are statistically significant and must be added to the model (up to that maximum number). This enables the user to test that an apparent change in trend is statistically significant [21]. The grid search method (GSM) was used to identify the joinpoints. Further, the Bayesian information criterion (BIC) was used to select the models that were best fitted [22]. The APC for each line segment and AAPC for the whole line segment with 95% confidence intervals is calculated by the software, and a Monte Carlo permutation method is used for significance test [23,24]. 

In the present report, due to the non-normal distribution of prevalence data, the log-linear model for trend analysis was used. The response variable was the natural logarithm of the annual infection rates, and the independent variable was the year. The association of infection rates with socioeconomic and sanitary conditions parameters was also analyzed in the study using the Pearson Correlation Coefficient. *p* < 0.05 was considered as statistically significant.

## 3. Results

### 3.1. Prevalence of Schistosomiasis in 2019

Over the 30-year interval studied, changes in infection rates of schistosomiasis were observed by sex and age. ASR per 100,000 for different gender and age in 2019 are shown in Table 1. In 2019, the mean ASR was 707.09 (621.43 to 929.13), and the mean ASR for women and men were 628.37 per 100,000 and 782.99 per 100,000, respectively. The highest ASRs were observed in the 25–29 years of age group (907.46, 708.06 to 1187.07). The lowest ASR was observed in <5 years of age group (75.19, 50.47 to 109.37). ASR for schistosomiasis in 2019 were higher in men than women.

### 3.2. Trends in Prevalence of Schistosomiasis

**Figure 1:**
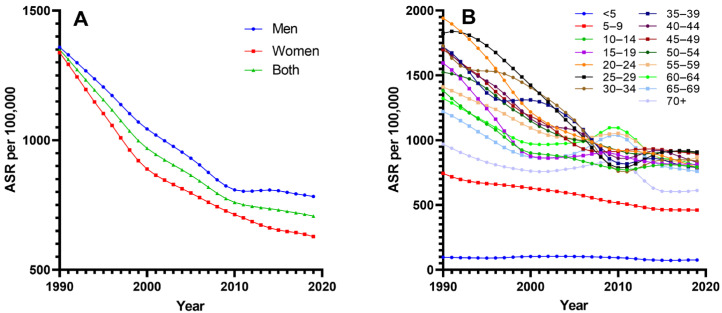
Trends in age-standardized prevalence rate per 100,000 of schistosomiasis between 1990 and 2019. (**A**) Men and women; (**B**) age groups; ASR: age-standardized infection rate (per 100,000).

Schistosomiasis ASR and its trends from 1990 to 2019 for different gender and age groups in China are presented in Table 1 and Figure 1. The ASR for both men and women is showing a decreasing trend, with a greater decline in females compared with males (men: −42.37, women: −53.05). There are 9 age groups (10 to 54 years old) of which the ASR has decreased by more than 40%, with the largest decline observed in the 25–29 age group (59.26%). The small decreases in ASR between 1990 and 2019 were observed in the <5 age group (22.72%).

Joinpoint analyses for ASR between 1990 and 2019 are shown in Table 2. Significant trend changes in ASR were identified in all age groups and both sexes. Prevalence trends were variable, but an overall downward trend was noted. The largest relative decrease in ASR for all were observed between 1994 and 2000, and the smallest between 2010 and 2019. The most rapidly decreasing trends were observed in the early and middle sections of the interval studied, most notably between 2005 and 2010 for the 25–29 age group, and between 1994 and 2000 for the 15–19 age group. The most rapidly decreasing trends for men were observed from 1995 to 1999, and for women were observed from 1994 to 2000.

Increases in schistosomiasis ASR were observed in several segments of a few age groups. The largest increases in ASR were noted in the trends covering the years 2006–2010 in the 60–64 age group. The age of 25–44 years mainly showed an upward trend between 2010 and 2015, and several age groups over 60 years of age showed the similar trend from the beginning of the 21st century to 2010.

### 3.3. Correlation between ASR and Socioeconomic, Sanitation Parameters

Table 3 displays the result of correlation analysis between ASR and GDP and PPMS for three separate time periods: 1990–2000, 2000–2010, and 2010–2019. The results show the schistosomiasis ASR is significantly correlated with GDP and PPMS in any given time period (*p* < 0.01). As is well-known, the decline of schistosomiasis ASR was closely related to the increase in the national socio-economy and rural sanitation usage.

## 4. Discussion

Based on the schistosomiasis prevalence data of China in GBD2019, this article uses the Joinpoint regression model to analyze the time changing trends of schistosomiasis prevalence in China from 1990 to 2019. It evaluates the characteristics of changes in schistosomiasis prevalence in different periods, genders, and age groups over the past 30 years, and analyzes the relationship between schistosomiasis prevalence and national economy, and the use of rural sanitation facilities. It provides a scientific basis for further optimizing the schistosomiasis control strategies in China and helps to promote the achievement of the national goal of schistosomiasis elimination [25].

The infection ASR of schistosomiasis shows different degrees of decline in different intervals. This trend was closely related to the implementation and adjustment of different schistosomiasis control strategies in China during this period. In the mid to late 1980s, schistosomiasis control strategy shifted from mainly snail control to morbidity control, especially after the World Bank-funded Schistosomiasis Control Project was launched in 1992 [26,27]. Large-scale chemotherapy, snail control in susceptible areas, and health education measures were implemented in the major endemic areas, leading to a continuous decline in the schistosomiasis incidence and number of cases [28,29]. However, with the end of the World Bank loan project in 2001, the funding for schistosomiasis control was insufficient, and the efforts for chemotherapy, snail control, and other measures decreased, leading to a serious rebound of the endemic situation in some areas, especially owing to the impact of the 1998 floods [30,31]. As a result, the decrease in ASR between 2000 and 2004 narrowed to 2.4%. After 2004, China adjusted its schistosomiasis control strategy to focus on controlling the infectious sources, with measures such as replacing cattle with machines, raising livestock in pens, improving fecal management, and providing access to safe water and sanitation. Meanwhile, the “National Mid and Long-term Plan for Schistosomiasis Control (2004–2015)” was implemented. The sanitation measures were popularization rural tap water and sanitary toilets, with the popularization rate reaching more than 90% in 2015; as for bovine, raising livestock in pens is the most crucial and required to reach 100% in all schistosomiasis-endemic countries in 2015, which leads to a significant reduction in the schistosomiasis incidence nationwide and meeting the epidemic control standards in 2008 [32,33,34]. Therefore, the decrease between 2004 and 2010 expanded again to 2.59%. After 2010, the decline narrowed to 0.73%, possibly due to the increased difficulty in compressing the epidemic after it decreased to a certain level. The ASR of schistosomiasis has shown an overall downward trend similarly in other countries such as Zimbabwe from 1990 to 2019, but in these countries, particularly in Africa, the infection rate remains high [35].

Gender is one of the factors influencing schistosome infection [36]. This study showed that the ASR for men has consistently been higher than that in women over the past 30 years, similar to previous findings [37]. The gap between male and female ASR gradually increased over time was also found. This may be due to the different modes of schistosome infection between genders, with men mainly infected through production activities such as fishing and shrimp catching, while women mainly are infected through daily activities such as laundry and washing. With the improvement of living standards and hygiene water conditions, daily activities are relatively easier to change compared with production activities, resulting in a greater decrease in ASR among women. These results suggest that effectively changing water contact behavior is of great significance for schistosomiasis control [38].

This study analyzed the trend of ASR among different age groups. The results showed that the ASR in all age groups decreased, with a relatively larger decline observed in 7 age groups from ages 15 to 49 with 5-year intervals. This may be related to the main occupational changes and differences in health education levels among individuals in this age group [39,40]. With the social and economic system changes, young and middle-aged farmers who were engaged in agriculture, fishing, and animal husbandry in rural areas have gradually migrated to cities for work and living, resulting in reduced chances of infection. For other age groups, due to their relatively fixed occupational and living environment, and low educational level in health, the decreasing trend was limited. A growth trend can be observed in 2 age groups from ages 60 to 69 between 2004 and 2010, mainly due to increased screening efforts by the Chinese government for this age group, which have revealed cases that were previously undetected. The research findings suggest that it is necessary to continue to pay attention to the prevention and control of schistosomiasis among people aged over 50 and under 15 years old. Combining the national strategy of rural revitalization, changing the rural industrial structure, and improving the schistosomiasis-prone environment will play a positive role in the prevention and control of schistosomiasis.

The prevalence of schistosomiasis is influenced by various factors such as natural environment, pathogenic organisms, and socioeconomics [41,42]. In this study, two socioeconomic indicators, namely per capita GDP and population coverage of safe sanitation facilities in rural areas, were selected to analyze their relationship with schistosomiasis. The results showed a significant negative correlation between the morbidity rate and per capita GDP, possibly due to increased investment in schistosomiasis control and improved awareness of residents’ health with economic development. In addition, the ASR was also significantly negatively correlated with the PPMS in rural areas, indicating that improving sanitation facilities and effectively managing human and livestock excrement are important measures for schistosomiasis control [43].

In this study, the infection data collected from GBD2019 were first used to analyze the prevalence of schistosomiasis in China, which solved the inconsistent indicators for measuring schistosomiasis infection over time. The schistosomiasis prevalent trend in China is overall reflected and similar to other studies. However, the data from the database were estimated from various literatures, not epidemiological surveys. Although the essential data disposing was applied to improve quality and comparability, uncertainty data accuracy and integrity make deviations difficult avoid in study.

Although the prevalence of schistosomiasis in China has been continuously decreasing and the epidemic is at a relatively low level, there are many challenges to overcome in order to achieve the goal of eliminating schistosomiasis. These challenges include a decrease in the level of attention, transmission through wild animals, an increase in leisure tourism in endemic areas, and more [44]. How to continue to promote the progress of schistosomiasis control is an important issue for successfully achieving the goal of eliminating schistosomiasis by 2030. According to the results of this study, the government’s attention and investment in schistosomiasis control have played an important role in reducing the disease incidence. The key to achieving the expected effect lies in adopting appropriate prevention and control strategies for different periods of schistosomiasis epidemics. Therefore, it is necessary to continue to adhere to the government-led mechanism for schistosomiasis control and implement strategies of precision prevention and control. At the same time, it is important to combine national development strategies such as rural revitalization, the construction of the Yangtze River Economic Belt, etc. By urbanization, economic development, improving rural health conditions, and changing the living environment of the population in endemic areas, the fundamental problem of schistosomiasis transmission can be fundamentally solved.

## 5. Conclusions

Due to continuous investment, optimization of prevention, and control strategies, economic and social development and improvement of rural health conditions, the prevalence of schistosomiasis continued to decline in China from 1990 to 2019. Schistosomiasis prevention is a systematic project. Only after the measures mentioned above are implemented is schistosomiasis elimination possible. The outcomes play instructive significance for further optimizing national integrated schistosomiasis control strategies to promote disease elimination.

## Figures and Tables

**Table 1:** The age-standardized infection rate from schistosomiasis in 1990 and 2019, and its change from 1990 to 2019.

Characteristics		ASR (95% UI), 1990	ASR (95% UI), 2019	Change Value, 1990–2019	Change Percentage, 1990–2019
Total		1349.55 (1146.35–1903.31)	707.09 (621.43–929.13)	642.46 (524.92–974.18)	47.61 (45.79–51.18)
Sex	male	1358.67 (1150.60–1919.85)	782.99 (685.74–1031.48)	575.68 (464.87–888.37)	42.37 (40.40–46.27)
	female	1338.43 (1142.10–1893.13)	628.37 (555.40–808.37)	710.07 (586.71–1084.76)	53.05 (51.37–57.30)
Age	<5	97.30 (42.84–195.48)	75.19 (50.47–109.37)	22.11 (−7.63–86.11)	22.72 (−17.80–44.05)
	5–9	743.28 (470.16–1177.23)	461.00 (330.97–620.99)	282.29 (139.19–556.24)	37.98 (29.60–47.25)
	10–14	1381.90 (898.55–2049.57)	793.04 (582.94–1062.67)	588.86 (315.60–986.90)	42.61 (35.12–48.15)
	15–19	1594.78 (1060.00–2312.52)	813.74 (616.87–1113.20)	781.04 (443.13–1199.31)	48.97 (41.80–51.86)
	20–24	1940.93 (1395.68–2835.54)	790.77 (600.24–1063.67)	1150.16 (795.44–1771.87)	59.26 (56.99–62.49)
	25–29	1824.70 (1402.95–2591.64)	907.46 (708.06–1187.07)	917.24 (694.89–1404.57)	50.27 (49.53–54.20)
	30–34	1727.47 (1287.28–2426.92)	839.54 (656.00–1114.66)	887.94 (631.28–1312.26)	51.40 (49.04–54.07)
	35–39	1719.96 (1395.59–2428.44)	788.68 (637.94–1050.40)	931.28 (757.65–1378.03)	54.15 (54.29–56.75)
	40–44	1719.72 (1349.48–2498.32)	829.23 (656.49–1103.03)	890.49 (692.99–1395.29)	51.78 (51.35–55.85)
	45–49	1698.29 (1389.96–2385.33)	895.87 (744.42–1168.40)	802.42 (645.54–1216.93)	47.25 (46.44–51.02)
	50–54	1524.51 (1260.89–2118.96)	904.08 (762.07–1170.62)	620.42 (498.82–948.35)	40.70 (39.56–44.76)
	55–59	1411.01 (1096.41–1944.08)	865.09 (702.68–1140.23)	545.92 (393.73–803.85)	38.69 (35.91–41.35)
	60–64	1323.04 (1000.82–1819.36)	801.92 (634.73–1048.41)	521.12 (366.09–770.95)	39.39 (36.58–42.37)
	65–69	1220.82 (980.84–1731.00)	759.47 (631.25–986.80)	461.35 (349.60–744.20)	37.79 (35.64–42.99)
	70+	969.16 (770.83–1382.70)	612.94 (512.32–807.15)	356.22 (258.51–575.55)	36.76 (33.54–41.62)

ASR: age-standardized infection rate (per 100,000); UI: uncertainty interval.

**Table 2:** Joinpoint analysis age-standardized infection rates from schistosomiasis in China by sex and age, 1990–2017.

Characteristics		Trend 1		Trend 2		Trend 3		Trend 4		Trend 5		Trend 6		Full Range
		Years	APC	Years	APC	Years	APC	Years	APC	Years	APC	Years	APC	AAPC
Total		1990–1994	−2.99 *	1994–2000	−3.50 *	2000–2004	−2.14 *	2004–2010	−2.59 *	2010–2019	−0.73 *			−2.20 *
Sex	male	1990–1995	−2.35 *	1995–1999	−2.99 *	1999–2005	−2.30 *	2005–2010	−2.92 *	2010–2014	0.20 *	2014–2019	−0.63 *	−1.89 *
	female	1990–1994	−3.75 *	1994–2000	−4.29 *	2000–2010	−2.13 *	2010–2014	−1.88 *	2014–2019	−0.96 *			−2.57 *
Age	<5	1990–1995	−1.58 *	1995–2000	2.83 *	2000–2005	0.17	2005–2010	−2.04 *	2010–2015	−5.12 *	2015–2019	1.11 *	−0.87 *
	5–9	1990–1993	−2.84 *	1993–1998	−0.96 *	1998–2005	−1.37 *	2005–2009	−2.70 *	2009–2015	−2.12 *	2015–2019	−0.08	−1.62 *
	10–14	1990–1996	−4.00 *	1996–1999	−5.14 *	1999–2005	−1.16 *	2005–2010	−2.16 *	2010–2015	1.08 *	2015–2019	−0.46 *	−1.87 *
	15–19	1990–1994	−4.57 *	1994–2000	−7.10 *	2000–2009	0.54 *	2009–2014	−1.78 *	2014–2019	−0.25			−2.33 *
	20–24	1990–1995	−3.34 *	1995–2000	−6.00 *	2000–2003	−3.37 *	2003–2009	−2.57 *	2009–2015	−0.92 *	2015–2019	−2.78 *	−3.08 *
	25–29	1990–1995	−0.99 *	1995–2003	−4.25 *	2003–2010	−6.64 *	2010–2015	3.50 *	2015–2019	0.02			−2.40 *
	30–34	1990–1992	−4.78 *	1992–1998	−0.64 *	1998–2005	−3.49 *	2005–2010	−8.73 *	2010–2015	2.77 *	2015–2019	−0.24	−2.43 *
	35–39	1990–1996	−4.65 *	1996–2001	−0.07	2001–2005	−3.08 *	2005–2010	−6.88 *	2010–2014	1.68 *	2014–2019	−1.90 *	−2.72 *
	40–44	1990–1997	−3.24 *	1997–2001	−4.82 *	2001–2005	−0.61 *	2005–2010	−4.72 *	2010–2015	1.60 *	2015–2019	−2.71 *	−2.47 *
	45–49	1990–1994	−3.02 *	1994–1998	−4.33 *	1998–2001	−3.02 *	2001–2007	−3.95 *	2007–2014	0.49 *	2014–2019	−0.88 *	−2.19 *
	50–54	1990–1994	−1.37 *	1994–2002	−3.78 *	2002–2012	−1.76 *	2012–2017	0.54 *	2017–2019	−0.6			−1.80 *
	55–59	1990–1996	−2.18 *	1996–2002	−2.92 *	2002–2005	−0.81	2005–2011	0.65 *	2011–2015	−5.59 *	2015–2019	0.64 *	−1.71 *
	60–64	1990–2000	−3.19 *	2000–2006	0.44 *	2006–2010	3.18 *	2010–2014	−5.78 *	2014–2019	−1.72 *			−1.70 *
	65–69	1990–2000	−3.56 *	2000–2004	0.59	2004–2010	3.15 *	2010–2015	−5.52 *	2015–2019	−1.04 *			−1.64 *
	70+	1990–1997	−2.86 *	1997–2002	−0.87 *	2002–2010	1.53 *	2010–2015	−6.76 *	2015–2019	0.47 *			−1.56 *

APC: Annual percentage change. AAPC: Average annual percentage change. * *p* < 0.050 versus 0 (output from joinpoint regression analysis).

**Table 3:** Correlation analysis of ASR with socioeconomic and sanitation parameters in China.

Time Periods	Index	ASR	
		r	*p* Value
1990–2019	GDP	−0.799 **	<0.01
	PPMS	−0.892 **	<0.01
1990–1999	GDP	−0.978 **	<0.01
	PPMS	NA	-
2000–2009	GDP	−0.956 **	<0.01
	PPMS	−0.985 **	<0.01
2010–2019	GDP	−0.991 **	<0.01
	PPMS	−0.994 **	<0.01

GDP: Gross national product per capita; PPSM: percentage of people using safely managed sanitation services; r: Pearson Correlation Coefficient; ** indicates a significant correlation at the 0.01 level (two-tail). NA: not available.

## Data Availability

The data used in the study are available from the corresponding author upon reasonable request.

## References

[1] Ogongo P., Nyakundi R.K., Chege G.K., Ochola L. (2022). The Road to Elimination: Current State of Schistosomiasis Research and Progress Towards the End Game. Front. Immunol..

[2] McManus D.P., Bergquist R., Cai P., Ranasinghe S., Tebeje B.M., You H. (2020). Schistosomiasis—From Immunopathology to Vaccines. Semin. Immunopathol..

[3] Gryseels B., Polman K., Clerinx J., Kestens L. (2006). Human Schistosomiasis. Lancet.

[4] Wiegand R.E., Fleming F.M., de Vlas S.J., Odiere M.R., Kinung’hi S., King C.H., Evans D., French M.D., Montgomery S.P., Straily A. (2022). Defining Elimination as a Public Health Problem for Schistosomiasis Control Programmes: Beyond Prevalence of Heavy-Intensity Infections. Lancet Glob. Health.

[5] Colley D.G., Bustinduy A.L., Secor W.E., King C.H. (2014). Human Schistosomiasis. Lancet.

[6] Vos T., Lim S.S., Abbafati C., Abbas K.M., Abbasi M., Abbasifard M., Abbasi-Kangevari M., Abbastabar H., Abd-Allah F., Abdelalim A. (2020). Global Burden of 369 Diseases and Injuries in 204 Countries and Territories, 1990–2019: A Systematic Analysis for the Global Burden of Disease Study 2019. Lancet.

[7] King C.H., Dickman K., Tisch D.J. (2005). Reassessment of the Cost of Chronic Helmintic Infection: A Meta-Analysis of Disability-Related Outcomes in Endemic Schistosomiasis. Lancet.

[8] Utzinger J., Raso G., Brooker S., De Savigny D., Tanner M., Ornbjerg N., Singer B.H., N’goran E.K. (2009). Schistosomiasis and Neglected Tropical Diseases: Towards Integrated and Sustainable Control and a Word of Caution. Parasitology.

[9] McManus D.P., Dunne D.W., Sacko M., Utzinger J., Vennervald B.J., Zhou X.-N. (2018). Schistosomiasis. Nat. Rev. Dis. Primers.

[10] Zhou X.-N., Wang L.-Y., Chen M.-G., Wu X.-H., Jiang Q.-W., Chen X.-Y., Zheng J., Utzinger J. (2005). The Public Health Significance and Control of Schistosomiasis in China—Then and Now. Acta Trop..

[11] Zhou Y., Chen Y., Jiang Q. (2021). History of Human Schistosomiasis (Bilharziasis) in China: From Discovery to Elimination. Acta Parasitol..

[12] Qian M.-B., Chen J., Bergquist R., Li Z.-J., Li S.-Z., Xiao N., Utzinger J., Zhou X.-N. (2019). Neglected Tropical Diseases in the People’s Republic of China: Progress towards Elimination. Infect. Dis. Poverty.

[13] Zhang L.J., Xu Z.M., Yang F., He J.Y., Dang H., Li Y.L., Cao C.L., Xu J., Li S.Z., Zhou X.N. (2022). Progress of schistosomiasis control in People’s Republic of China in 2021. Chin. J. Schistosomasis Control.

[14] Abe E.M., Tambo E., Xue J., Xu J., Ekpo U.F., Rollinson D., Yang K., Li S.-Z., Zhou X.-N. (2020). Approaches in Scaling up Schistosomiasis Intervention towards Transmission Elimination in Africa: Leveraging from the Chinese Experience and Lessons. Acta Trop..

[15] Feng J., Zhang X., Hu H., Gong Y., Luo Z., Xue J., Cao C., Xu J., Li S. (2023). Spatiotemporal Distribution of Schistosomiasis Transmission Risk in Jiangling County, Hubei Province, P.R. China. PLoS Negl. Trop. Dis..

[16] Xiao Y., Zhong C.H., Wei F.H., Dai L.F., Yang J.J., Chen Y.Y. (2022). Epidemiological trends for human schistosomiasis prevalence in Hubei Province from 2004 to 2018 based on Joinpoint regression analysis. Chin. J. Schistosomasis Control.

[17] Wu X.H., Wu J., Xu R.M., Xiong Y., Chen Z. (2022). Epidemiological trends of schistosomiasis in Poyang county of Jiangxi Province from 2004 to 2020 based on the Joinpoint regression model. Chin. J. Schistosomasis Control.

[18] Li F.Y., Tan H.Z., Ren G.H., Jiang Q., Wang H.L. (2017). Research of prevalence of schistosomiasis in Hu Nan province, 1984–2015. Chin. J. Epidemiol..

[19] Zhou X.N., Wang T.P., Wang L.Y., Guo J.G., Yu Q., Xu J., Wang R.B., Chen C., Jia T.W. (2004). The current states of schistosomiasis epidemics in China. Chin. J. Epidemiol..

[20] Roth G.A., Abate D., Abate K.H., Abay S.M., Abbafati C., Abbasi N., Abbastabar H., Abd-Allah F., Abdela J., Abdelalim A. (2018). Global, Regional, and National Age-Sex-Specific Mortality for 282 Causes of Death in 195 Countries and Territories, 1980–2017: A Systematic Analysis for the Global Burden of Disease Study 2017. Lancet.

[21] Kim H.J., Chen H.S., Byrne J., Wheeler B., Feuer E.J. (2022). Twenty years since Joinpoint 1.0: Two major enhancements, their justification, and impact. Stat. Med..

[22] Vrieze S.I. (2012). Model selection and psychological theory: A discussion of the differences between the Akaike information criterion (AIC) and the Bayesian information criterion (BIC). Psychol. Methods.

[23] Clegg L.X., Hankey B.F., Tiwari R., Feuer E.J., Edwards B.K. (2009). Estimating average annual percent change in trend analysis. Stat. Med..

[24] Kim H., Fay M., Feuer E., Midthurne D. (2000). Permutation Tests for Joinpoint Regression with Applications to Cancer Rates. Stat. Med..

[25] The Central Committee of the Communist Party of China and the State Council Issued “Healthy China 2030” Program, Relevant Documents of the Central Government, Chinese Government Website. http://www.gov.cn/zhengce/2016-10/25/content_5124174.htm.

[26] Lin D.D., Wu H.W., Wu G.L., Zhou X.N. (2007). Review and evaluation on optimal combined strategies for schistosomiasis control in China. Chin. J. Schistosomasis Control.

[27] Li Y.F., Lin D.D. (2019). Process and prospect of schistosomiasis control in Jiangxi Province. J. Trop. Dis. Parasitol..

[28] Luo T.P., Li Y.L., Zhang X.Q., Fang W. (2002). Evaluation on world band load schistosomiasis control programme in Yunnan province from 1992–2000. Chin. J. Schistosomasis Control.

[29] Cai K.P., Li Y.Y., Li X.Y., Jiang Q. (2003). Effectiveness of world bank loan schistosomiasis control project in 9 years, in Hunan province. Chin. J. Schistosomasis Control.

[30] Wang R.B., Wang T.P., Wang L.Y., Guo J.G., Yu Q., Xu J., Gao F.H., Yin Z.C., Zhou X.N. (2004). Study on the re-emerging situation of schistosomiasis epidemics in areas already under control and interruption. Chin. J. Epidemiol..

[31] Lin D.D., Hu F., Liu Y.M., Chen H.G. (2004). Analysis of Schistosomiasis Epidemic Situation after World Bank Loan in Poyang Lake District and Its Control Countermeasures. Chin. J. Epidemiol..

[32] Wang L.-D., Chen H.-G., Guo J.-G., Zeng X.-J., Hong X.-L., Xiong J.-J., Wu X.-H., Wang X.-H., Wang L.-Y., Xia G. (2009). A Strategy to Control Transmission of Schistosomasis Japonicum in China. N. Engl. J. Med..

[33] Li Y.F., Hang C.Q., Hu F., Yuan M., Gu X.N., Lu S.B., Zeng X.J., Lin D.D. (2017). Role of new strategy in transmission control of schistosomiasis in Poyang Lake region. Chin. J. Schistosomasis Control.

[34] Wang L.D., Zhou X.N., Chen H.G., Guo J.G., Zeng X.J., Hong X.L., Xiong J.J., Wu X.H., Wang L.Y., Xia G. (2009). A new strategy to control transmission of Schistosomasis japonicum. Strateg. Study CAE.

[35] Li H.M., Zheng J.X., Qian Y.J., Lv S., Xia S., Zhou X.N. (2023). Comparison of the disease burden of schistosomiasis globally and in China and Zimbabwe. Chin. J. Schistosomasis Control.

[36] LoVerde P.T. (2019). Schistosomiasis. Adv. Exp. Med. Biol..

[37] Dong X.Q., Feng X.G., Dong Y., Xiong M.T., Jiang H., Shen M.F., Zhang Y., Yang G.C., Shao Z.T., Wu X. (2008). Epidemiological characteristics and control strategies of schistosomiasis in mountainous areas of Yunnan Province. Chin. J. Schistosomasis Control.

[38] Kloos H. (1995). Human Behavior, Health education and schistosomiasis control: A Review. Soc. Sci. Med..

[39] He J.C., Zheng B.Q., Fang G.R., Wan S.K., Zhang X.G., Xia C.G., Wang Q.Z., Dong X.Z., Wu W.Z., Ge J.H. (2000). Evaluation on the cost-effectiveness of two chemotherapy strategies for schistosomiasis control in medium endemic area. Chin. J. Parasit. Dis. Con..

[40] Feng J.X., Gong Y.F., Luo Z.W., Wang X.Y., Cao C.L., Zhang X., Zhang J.F., Li H.R., Hu H.H., Xu J. (2022). A survey on the current situation of schistosomiasis prevention and control knowledge and belief among adults in Jiangling county. Mod. Prev. Med..

[41] Engels D., Zhou X.-N. (2020). Neglected Tropical Diseases: An Effective Global Response to Local Poverty-Related Disease Priorities. Infect. Dis. Poverty.

[42] Zheng J. (2009). Achievements and challenges in schistosomiasis control in China. Chin. J. Parasitol. Parasit. Dis. Oct..

[43] Wang L.D. (2005). The key to controlling the prevalence of schistosomiasis in China is to manage human and animal faeces well. Chin. J. Epidemiol..

[44] Miranda G.S., Rodrigues J.G.M., Silva J.K.A.d.O., Camelo G.M.A., Silva-Souza N., Neves R.H., Machado-Silva J.R., Negrão-Corrêa D.A. (2022). New Challenges for the Control of Human Schistosomiasis: The Possible Impact of Wild Rodents in Schistosomasis Mansoni Transmission. Acta Trop..

